# Screening Isn’t Enough: A Call to Integrate Behavioral Health Providers in Women’s Health and Perinatal Care Settings

**DOI:** 10.5334/ijic.5640

**Published:** 2020-11-18

**Authors:** Cara M. C. English

**Affiliations:** 1 Cummings Graduate Institute for Behavioral Health Studies, US; 2 Terra’s Place for Maternal Mental Health, US

**Keywords:** integrated care, perinatal mental health, antenatal, value creation

## Abstract

This paper aims to enhance the current understanding of integrated mental health services in the United States and how they can be better incorporated in perinatal and women’s health specialty care from the perspective of a behavioral health provider. While much is known about gender disparities of mental health and low recognition and treatment rates for mental health disorders in women’s health and perinatal care, few changes are being implemented to embed mental health specialists where they are needed most to close gaps in care. This paper demonstrates the value creation of integrated behavioral health in a midwife practice in the State of Arizona. Clinical and operational workflows can easily be adapted to include a behavioral health service to address mental and behavioral health needs that, when untreated, lead to long-term adverse outcomes in women and their families. Financial barriers that may hinder success of the integrated model are highlighted and discussed.

## Introduction and Definitions

In the United States, a key aim of integration efforts has been to embed mental health and social care practitioners (hereafter referred to as “behavioral health providers”) in primary care settings to increase identification and treatment of mental & behavioral components of chief complaints and chronic disease. In this paper, “behavioral health” refers to the roles thinking, feeling, and behaving play in wellness and chronic disease. Furthermore, in this paper, “integrated care” refers to on-site teamwork with a unified care plan involving combined interventions for mental health disorders, organizational structure that emphasizes staff communication, links to community social programs to address all patient needs, and unified billing to third party or public health insurance plans [11].

## What Are Behavioral Health Providers in the United States?

Behavioral health providers (BHPs) use evidence-based interventions to increase awareness of how thoughts, feelings, and behaviors can impact common chief complaints, such as insomnia, and to increase patient motivation to take action to improve health. BHPs offer training and consultation to the medical team and to patients and families, screen patients for common mental health illnesses including childhood trauma, depression, anxiety, and bipolar disorder; common behavioral disorders such as self-harm, substance abuse, eating disorders, physical inactivity, nutritional deficiencies, and sleep disorders; and assist physicians in choosing appropriate psychiatric medications when necessary to improve mental health and increase behaviors that support better health. Additionally, BHPs assist medical teams in assessing psychosocial disparities such as financial instability, unsafe housing, transportation, food insecurity, domestic violence, occupational disruption, and gaps in care due to racism, gender, sexual orientation, or other large social disparities which are persistent in the US [4,13,14]. It is estimated that less than 50% of patients referred to mental health specialty care in the US initiate the care they need [3]. By contrast, when behavioral health care is integrated in primary care settings, initiation rates have been measured at up to 95.5% [3,17].

## Gender Disparities

Gender specific risk factors for mental health disorders disproportionately impact women [20]. It is estimated that women in the US experience a 1.5–2.5 times greater lifetime prevalence of depression and anxiety compared to males [18]. While perinatal mood and anxiety disorders (PMADs) are common in pregnancy and in the year following birth, affecting 15%–34% of women annually, recognition and treatment rates are lower in pregnant and postpartum women than in the general population [19]. Tragically, the long-term overall risk of suicide in pregnant women is 17 times that of the general female population and it is 70 times higher during the first year postpartum [18]. Suicide is the second leading cause of death in postpartum women [12,19]. Antenatal and postpartum depression increase the risk for multiple adverse outcomes among women and their children, interfere with proper infant attachment and child development, and increase rates of poor cognitive performance [12]. These data point to a clear need for behavioral health integration in women’s health and perinatal care specialties.

The COVID-19 pandemic has significantly increased the experience of anxiety and depression in pregnant and postpartum mothers, who are facing birthing in hospitals without partners or hired labor support, disease contraction fears, and personal and professional challenges including job losses that often result in losses of health insurance coverage for the entire family. A recent study estimates that while rates of anxiety and depression were relatively high among pregnant women and women in the 1st year after delivery prior to the pandemic (29% and 15%, respectively), 72% of women currently meet criteria for moderate to high anxiety and 40.7% of these women meet criteria for moderate depression [6]. From gender disparities in primary child caregiver (44% of women vs. 14% of men) to accepting the burden of home-schooling children or supervising e-learning from home on top of working from home, 74% of US mothers reportedly feel mentally worse since the pandemic began [10]. These data support the urgent need for integration efforts in response to the new mental health pandemic, secondary to the COVID-19 pandemic.

We know that untreated mental illness increases the risk for multiple adverse outcomes among women and their children, interferes with infant attachment and child development, and increases rates of poor cognitive performance [8,12]. Globally, at least 86% of women receive prenatal care with a skilled health professional [16], making perinatal care a key target for mental health integration; however, this type of integration is not prevalent in the State of Arizona or in the United States, in general.

So, what if we knew what change we needed to make, and actually did it? What if that change significantly and positively impacted the healthcare experience of pregnant and postpartum women, and that impact generalized to their immediate and sometimes even extended family members? In a world turned upside down, *what if we could share some good news*?

## Context and Aims of the Integration Effort

I am a Doctor of Behavioral Health (DBH), a degree which was created in 2009 to fulfill the unmet educational need to prepare BHPs to work in integrated medical settings; shoulder-to-shoulder with interprofessional medical teams. To address the need for perinatal mental health services, a partnership was formed in 2017 between Willow Midwife Center for Birth and Wellness and my outpatient behavioral health practice, Terra’s Place for Maternal Mental Health and Wellness, both located in the metropolitan suburbs of Phoenix, Arizona, US. Leveraging evidence-based guidelines for screening, assessment, and treatment, the integrated Willow team consisted of myself, a part-time BHP with advanced training and expertise in integrated perinatal mental health, and five full-time certified nurse midwives. Together, we made the primary women’s health care and perinatal care practice whole. While many systems are reluctant to integrate mental health into primary care due to the initial costs and workflow adjustments, our team worked quickly to address the challenges of integration.

## The Change We Made

Willow midwives were already using the American College of Obstetricians and Gynecologists (ACOG) recommended screening tool for perinatal depression, the Edinburgh Postnatal Depression Scale (EPDS) [2,5,9] at 36 weeks gestation and again at 6 weeks postpartum; earlier in care if clinically indicated. Upon launching our partnership, women with a score ≥10 on the EPDS were referred for a behavioral health consultation. Over the initial 3-month period of our partnership in 2017, we implemented additional screening tools (Adverse Childhood Experiences (ACE) & Mood Disorder Questionnaire (MDQ)) to provide metrics for measuring severity of illness and response to treatment over time and to differentiate between unipolar and bipolar depression, as an estimated 60% of bipolar illness is misdiagnosed as unipolar depression in perinatal women [12,1,7]. During well woman, prenatal, and postnatal visits, midwives used clinical judgement to identify stress and to invite women to schedule a behavioral health appointment. They referred to me as “Dr. English, our behavioral health provider,” and the service was introduced at “Meet & Greet” visits in the birth center to reduce stigma and level set expectations for care to include behavioral health. During initial behavioral health visits, ACE, EPDS, and MDQ screening was followed by an evidence-based clinical interview to explore psychosocial needs and disparities in the same environment in which they received prenatal or primary care. A treatment plan was discussed with women during their initial behavioral health consultation, and this was shared with the clinical team to ensure the team worked in partnership on all treatment goals. When medication was clinically recommended for anxiety or unipolar depression, a prescription was initiated by the midwife team. When bipolar illness was indicated based on screening results and results of the clinical interview, a referral to a psychiatric mental health nurse practitioner specializing in perinatal psychiatry was coordinated, and communication with that provider was part of the treatment plan. The shared electronic health record assisted all providers in remaining informed about the treatment and medications prescribed at all times.

## Results

### Patients

Willow patients were surveyed on satisfaction with care and wellness outcomes. Survey results indicated that the integrated behavioral health service greatly improved the perinatal experience for 73.9% of Willow patients, and that 91% preferred behavioral health to be integrated in perinatal/women’s health care. One hundred percent (100%) of those surveyed indicated that they had access to behavioral health care when they needed it and that they achieved resolution for their presenting concern. EPDS data indicated that on average, stress due to mental health disorders was reduced to a “mild” (<9) level in 6 sessions or less. Women who had higher ACE scores (≥4), those with a history of significant and serious mental illness, and those with significant marital or partner conflict typically needed more care and/or a referral to specialty providers or community resources to resolve mental health stress. A need for an atlas of social programs available in the community was identified and plans to implement social prescribing and case management to meet needs are underway. While a large percentage of women screened positive for self-harm ideation on the EPDS, we successfully used an additional screen for risk and protective factors [15] to determine action steps and effective treatment plans.

### Providers

One hundred percent of Willow providers indicated that the impact of behavioral health integration was very positive, and that the behavioral health training was helpful. Most midwives (60%) indicated that they consulted with the BHP more than once a week and the other 40% indicated they consulted with the BHP at least once a week. The medical team was less fearful of using screening tools due to the access of an in-house BHP. Midwives were happy with the behavioral health consultation and training they were receiving and felt that they could focus more on midwifery care, and not worry so much about having to treat behavioral/mental illness. Some midwife interns stated that they competed aggressively for a full-time position at Willow after completing their training and licensure because of the behavioral health service. These midwives stated that they had completed internship hours at sites without a BHP and did not wish to work in such a setting.

### Payors

While billing is always a challenge in the United States, and we have worked closely with medical billers and insurance companies to ensure that claims are paid in a timely manner, all behavioral health claims were paid. Patient out-of-pocket costs for care varied by patient and by plan. Arrangements were made with patients to ensure affordability of care or referrals to grant-funded services in the community when necessary; however, this was a need for a very small number of our population. A complete return on investment analysis has yet to be calculated.

To date, we have been an integrated team for three years. To meet the growing interest in and demand for integrated mental health services, we hired a second BHP in 2018 and will be hiring a full-time BHP in 2020 to support the second location Willow opened in 2019.

## Discussion & Key Lessons Learned

The largest challenge in this integration effort is the quagmire of the US health insurance system. For each woman who seeks care, benefits are verified and communicated to the patient by the Willow administrator, so that she is aware of her out-of-pocket costs for her care. Unfortunately, in the US, behavioral health is still reimbursed through a carve-out model, which causes significant delays in reimbursement, significantly lower reimbursement rates for BHPs than for medical providers, and high copay, coinsurance, and deductible costs for our patients. This financial burden for providers causes burnout, large overhead costs, and threatens the business model. Simultaneously for patients, the out-of-pocket costs causes significant stress, undoing some of the good work that behavioral health visits have achieved. We have yet to see a value-based care or bundled prenatal/postnatal package that includes adequate reimbursement for behavioral health care; however, we intend to leverage our good outcomes to negotiate for better reimbursement from insurance payors in 2021. Should we achieve this goal, we will solve the remaining challenge standing in the way of generalizing this model of care more widely in our state and beyond.

It should be noted that this integration effort may owe much of its success to the skills and training of the Doctor of Behavioral Health, whose doctoral education focused exclusively on integrating mental health in medical settings. From a workforce perspective, identifying BHPs with the right training and skill set will be critical, as the BHP must be prepared to lead with a firm grasp of Peek’s Three World Concept of Behavioral Health Integration [11]: clinical, operational, and financial “worlds” must be addressed simultaneously to achieve success.

## Conclusion

Adopting an integrated perinatal and women’s health care model can resolve mental and behavioral health disorders in women and prevent adverse long-term outcomes for women and families. While healthcare systems vary across countries, the international network benefits from strengthening our understanding of successes in different contexts. The COVID-19 pandemic has put a spotlight on the global need for mental health and social services; particularly for women, but our health delivery systems are not yet prepared to meet these needs or to disrupt the poor outcomes for our most vulnerable community members. This paper demonstrates specific workflow changes that can be effectively implemented in any country to serve goals for population health beyond routine care activities, and to create value for people, providers, and payors. Also highlighted are workforce education and training needs that must be considered to ensure success of implementation. Our hope is that sharing our experiences will inform and inspire international adoption of integrated perinatal behavioral health care.
